# Effect and Safety of Bronchoscopy Based on Imaging Evaluation in Patients With Acute Exacerbation of Bronchiectasis

**DOI:** 10.1155/carj/3117887

**Published:** 2026-06-04

**Authors:** Biliang Li, Zeng Juan, Mingfeng Wang, Wei Kang, Bo Li

**Affiliations:** ^1^ Department of Respiratory and Critical Care Medicine, Bazhong Central Hospital, Bazhong, 636000, Sichuan, China, scu.edu.cn

**Keywords:** acute exacerbation, bronchiectasis, bronchoscopy interventional technology, imaging evaluation, safety

## Abstract

**Objective:**

To evaluate the efficacy and safety of imaging‐assisted bronchoscopic interventions in acute bronchiectasis exacerbations and analyze their effects on pulmonary function, inflammation, and quality of life for evidence‐based clinical support.

**Methods:**

A total of 80 inpatients with acute bronchiectasis exacerbations were randomized into a control group (*n* = 40), receiving conventional anti‐infective and symptomatic treatment, and an intervention group (*n* = 40), receiving additional imaging‐assisted bronchoscopic intervention. Pulmonary function, inflammatory markers (C‐reactive protein [CRP], procalcitonin [PCT], and white blood cell count [WBC]), and St George’s Respiratory Questionnaire (SGRQ) quality of life scores were assessed preintervention and at 1, 2, and 4 weeks. Adverse events (e.g., hemoptysis, pneumothorax, and infection spread) and overall effectiveness were recorded.

**Results:**

No significant intergroup differences were observed in baseline characteristics, preintervention pulmonary function (forced expiratory volume in the first second [FEV_1_], forced vital capacity [FVC], and FEV_1_/FVC), inflammatory markers (CRP, PCT, and WBC), or SGRQ scores (*p* > 0.05). After 1, 2, and 4 weeks, the intervention group demonstrated significantly greater improvements: higher FEV_1_, lower CRP and PCT levels, and reduced SGRQ scores compared with the control group (*p* < 0.05). The generalized estimation equation (GEE) model revealed that the improvement magnitude in the intervention group increased over time (*p* < 0.001). The overall adverse event rate in the intervention group was 10.00% (4/40), significantly lower than the control group’s 37.50% (15/40); the total effective rate was 92.50% (37/40), surpassing the control group’s 70.00% (28/40) (*p* < 0.05).

**Conclusion:**

Bronchoscopy intervention guided by imaging improves lung function, reduces inflammation and adverse events, enhances quality of life, and demonstrates good safety with high clinical value in acute bronchiectasis exacerbations. The therapeutic benefit is likely attributable to the bronchoscopic procedure itself, including targeted pathogen identification and subsequent antibiotic modification, rather than solely to transient sputum clearance.

## 1. Introduction

Bronchiectasis, a respiratory disorder characterized by irreversible bronchial wall dilation and chronic infection, often presents with acute exacerbations marked by airway mucus obstruction, excessive purulent secretions, and uncontrolled inflammation. These manifestations significantly worsen symptoms including coughing, sputum production, and dyspnea, potentially progressing to respiratory failure or multiorgan dysfunction in severe cases [1]. Clinical data indicate that approximately 60% of the patients experience at least one acute exacerbation annually, with recurrent episodes accelerating pulmonary decline and reducing 5‐year survival rates to 50%–70% [2]. The pathogenesis of bronchiectasis involves a vicious vortex of airway infection, bronchial structural distortion, mucociliary clearance impairment, and immune dysregulation, which collectively drive disease progression and recurrent exacerbations. Current mainstream interventions focus on systemic anti‐infection therapy, expectorant administration, and symptomatic support. However, for patients with localized lesions and structural airway obstruction, medications struggle to reach the core affected areas, resulting in intervention response rates below 40% [3, 4]. Bronchoscopic interventional techniques offer a novel approach to break the pathogenetic vicious vortex by directly clearing airway mucus and phlegm and administering local medications. Nevertheless, traditional bronchoscopy heavily relies on operator experience for lesion localization, leading to high uncertainty and incomplete lesion clearance, with postoperative complication rates reaching 25%–30% [5, 6]. Recent advancements in chest high‐resolution computed tomography (HRCT) 3D reconstruction have enabled precise visualization of bronchiectasis location, extent, and lumen morphology, providing objective anatomical evidence for interventional therapy [7]. Previous studies have demonstrated the potential value of image‐guided bronchoscopic interventions in patients with chronic stable‐phase bronchiectasis, including improved airway clearance and reduced bacterial burden [8, 9]. However, high‐level evidence‐based studies remain scarce regarding the application of this approach during acute exacerbations, particularly in terms of efficacy validation, dynamic indicator analysis, and safety assessment. Bronchiectasis is a highly heterogeneous disease, and the underlying etiology is a key determinant of natural history and prognosis; therefore, understanding the specific role of bronchoscopy across different clinical scenarios is essential. We hypothesized that HRCT‐guided bronchoscopic intervention during acute exacerbations would result in superior improvements in pulmonary function, inflammatory control, and quality of life compared with conventional treatment alone, with an acceptable safety profile.

## 2. Data and Methods

### 2.1. General Information

This study enrolled 80 patients with acute exacerbations of bronchiectasis who visited the Department of Respiratory Medicine at our hospital between January 2020 and April 2025. The protocol was approved by the Medical Ethics Committee of our hospital (approval no.: 2019–086), with all patients or their legal guardians signing written informed consent. This study was not prospectively registered in an international trial registry, which is acknowledged as a limitation.

1. Inclusion criteria are as follows: (1) Meet diagnostic criteria for bronchiectasis [10], confirmed by chest HRCT showing bronchiectasis signs; (2) be in acute exacerbation phase, defined as a clinical deterioration with at least three of the following for at least 48 h: increasing cough, increasing sputum volume or purulence, worsening dyspnea, hemoptysis, and/or fever, based on the British Thoracic Society guidelines [10]; (3) age 18–75 years; (4) forced expiratory volume in the first second (FEV1) ≤ 30% of predicted value on pulmonary function tests, which was selected to include patients with moderate‐to‐severe airflow limitation who would most benefit from bronchoscopic intervention; (5) expected survival period ≥ 6 months; and (6) patient and family members cooperate with interventions and follow‐up.
2. Exclusion criteria are as follows: (1) Severe cardiopulmonary insufficiency or hepatic/renal failure; (2) coagulation disorders (international standardized ratio of prothrombin time > 1.5); (3) severe pulmonary hypertension (PA systolic pressure > 50 mmHg); (4) bronchoscopic contraindications (e.g., severe laryngeal edema and aortic aneurysm); (5) allergy to local anesthetics or medications used in interventional procedures; (6) received bronchoscopic intervention within 1 month; and (7) comorbidities such as lung cancer or tuberculosis.

The randomization was conducted using the digital table method: Statistical specialists generated a sequence of 1–80 random numbers, which were assigned sequentially to patients based on their enrollment order. Odd‐numbered individuals were allocated to the intervention group, while even‐numbered individuals were assigned to the control group, with each group containing 40 cases. The grouping process utilized a blinding envelope method to conceal the allocation information until data collection was completed and the blinded status was revealed. Sample size was calculated based on a two‐sided significance level of 0.05, power of 80%, and an expected difference of 5% in FEV1 between groups with an estimated standard deviation of 7%, yielding a minimum of 32 patients per group. To account for approximately 20% dropout, 40 patients were enrolled in each group.

### 2.2. Intervention Methods

#### 2.2.1. Basic Interventions

Both groups received standardized routine basic interventions, with specific protocols including (1) antimicrobial therapy: deep lung aspiration specimens were collected within 48 h of hospital admission for bacterial culture and drug sensitivity testing. Initial empirical broad‐spectrum antibiotics (e.g., cefoperazone–sulbactam sodium 2.0 g intravenous infusion every 12 h and levofloxacin 0.5 g intravenous infusion once daily) were administered. The regimen was adjusted to sensitive antibiotics based on drug sensitivity results, with a treatment duration of 10–14 days to ensure targeted antimicrobial therapy. (2) Sputum clearance therapy: sputum was graded based on its viscosity and characteristics to guide expectorant therapy: (1) Grade I (thin sputum and easily expectorated): administer ambroxol injection 30 mg intravenously twice daily; (2) Grade II (moderate viscous): combined with acetylcysteine nebulization (0.3 g added to 3 mL saline, oxygen‐driven nebulization, 15 min per session twice daily); (3) Grade III (purulent viscous sputum): add N‐acetylcysteine effervescent tablets 600 mg orally twice daily to promote sputum liquefaction and expulsion. (3) Symptomatic supportive therapy: fever patients received acetaminophen tablets 0.5 g orally when temperature ≥ 38.5°C (repeat every 4–6 h, maximum daily dose ≤ 2 g); Dyspneic patients received nasal cannula or mask oxygen therapy to maintain blood oxygen saturation at 90%–92%; For those with arterial oxygen partial pressure < 60 mmHg after oxygen therapy, immediate noninvasive ventilator assistance (S/T mode, suction pressure 8–12 cmH_2_O, and exhalation pressure 4–6 cm H_2_O) was provided while dynamically monitoring arterial blood gas parameters.

#### 2.2.2. Control Group

Only the above conventional intervention plan was used. During the intervention, the dosage and course of medication were adjusted according to the changes of patients’ symptoms but no bronchoscopy‐related interventional operation was performed. The nature and amount of sputum and the degree of dyspnea were recorded daily, and chest x‐ray was reviewed weekly to evaluate the absorption of inflammation.

#### 2.2.3. Intervention Group

Implementing bronchoscopic interventional therapy based on imaging assessment in addition to conventional interventions, specifically including the following:

1. Imaging evaluation: both groups underwent baseline chest imaging assessment. The intervention group received a comprehensive chest HRCT examination within 24 h prior to intervention (layer thickness 0.625 mm and screw pitch 1.0). The control group received standard chest x‐ray monitoring. The rationale for additional HRCT in the intervention group was to enable precise procedural planning, including multiplanar reconstruction (MPR) and volumetric reconstruction (VR) to accurately measure the lumen diameter, length, and branching angles of the affected bronchus. Determine the target areas (lobes/segment), extent (number of involved segments), and severity (graded by Reiff scoring system: each involved lobe was scored based on bronchiectatic morphology, with 1 point for cylindrical, 2 points for varicose, and 3 points for cystic bronchiectasis; the maximum total score was 18). Mark key targets such as mucus plugs and strictures, and generate visualized intervention pathway diagrams. In cases of diffuse bronchiectatic lesions involving multiple lobes, the most severely affected segments with the largest mucus plugs or the greatest degree of obstruction were prioritized for targeted intervention.
(2) Bronchoscopic intervention: (1) Preoperative preparation: complete coagulation function tests (prothrombin time and activated partial thromboplastin time) and electrocardiogram examination; fasting for 6 h; establish intravenous access with hemostatic agents (1.0 g tranexamic acid injection) and emergency equipment; and position the patient supine with 2% lidocaine gel (10 mL) for pharyngeal local anesthesia combined with cricothyroid membrane puncture (2 mL 2% lidocaine) to ensure adequate airway anesthesia. (2) Intraoperative procedure: insert electronic bronchoscope (Olympus BF‐1T260, 2.8 mm working channel) through nasal or oral route. Locate lesions using HRCT reconstruction images: for Grades I‐II mucous plugs, gently remove with biopsy forceps; for Grade III hard mucous plugs, use ERBECC‐450 cryoprobe at −70°C with three freeze‐thaw cycles before complete removal. Perform segmental irrigation with 20–30 mL 37°C saline, suctioned back by negative pressure (100–150 mmHg) for 3‐4 cycles. Total irrigation volume should not exceed 100 mL, with ≥ 50% irrigation fluid collected for pathogen testing (bacterial/fungal culture and drug sensitivity). Administer sensitive antibiotics (e.g., 0.5 g cefotaxime or 80 mg tobramycin diluted in 5 mL saline) at most affected sites, slowly injected followed by airway clamping for 5 min. (3) Postoperative management: patients are fasted for 2 h after surgery, with continuous monitoring of blood oxygen saturation and vital signs during this period. For minor hemoptysis (< 5 mL), Yunnan Baiyao capsules (0.5 g) are administered orally three times daily. Immediate intravenous infusion of 1.0 g of tranexamic acid is required if hemoptysis exceeds 10 mL. (4) Intervention schedule: the initial intervention should be completed within 48 h of diagnosis. A follow‐up HRCT scan on Day 7 assesses lesion changes. Patients with persistent mucus retention or uncontrolled inflammation will undergo a second intervention. A third intervention may be necessary after re‐evaluation on Day 14, with a maximum of three interventions to prevent excessive airway damage. The timing of the initial bronchoscopy within 48 h of diagnosis was chosen to maximize the benefit of early airway clearance during the acute inflammatory phase.
(3) Both groups received four consecutive weeks of standardized interventions. Daily symptom logs recorded cough frequency (coughs/h), sputum production (mL/24 h), and dyspnea scores (mMRC grading). Venous blood samples were collected every Tuesday and Friday for inflammatory marker testing. The final follow‐up was conducted 4 weeks after intervention to evaluate long‐term outcomes. All 80 enrolled patients completed the full 4‐week follow‐up period with no dropouts or losses to follow‐up. Patients remained hospitalized for the initial 2 weeks and were followed as outpatients for the remaining 2 weeks.

### 2.3. Observation Indicators

#### 2.3.1. Pulmonary Function Indicators

Pulmonary function tests were conducted using a pulmonary function instrument (Model: MasterScreen Pneumo) at baseline, 1 week, 2 weeks, and 4 weeks postintervention. The parameters included (1) FEV1 as a percentage of predicted value: fefined as the ratio of FEV1 to the predicted value for individuals of the same age, gender, and height. (2) Forced vital capacity (FVC): measured as the maximum volume of air expelled after maximal inhalation, expressed in liters (L). FVC was reported in absolute values rather than percent predicted to provide complementary information alongside FEV1% predicted, as both metrics together offer a more comprehensive assessment of pulmonary function.(3) FEV1/FVC ratio: calculated as the ratio of FEV1 to the FVC.

#### 2.3.2. Inflammatory Markers

(1) C‐reactive protein (CRP): measured using the immunoturbidimetric method with a normal range of 0–10 mg/L; (2) procalcitonin (PCT): assessed through electrochemiluminescence with a normal range < 0.05 ng/mL; and (3) white blood cell count (WBC): analyzed via automated hematology analyzers, with a normal range of (4–10) × 10^9^/μL. Fasting venous blood samples were collected before intervention and at 1, 2, and 4 weeks posttreatment for testing.

#### 2.3.3. Quality of Life Assessment

The St George’s Respiratory Questionnaire (SGRQ) [11] was employed to evaluate patients’ quality of life. This instrument comprises three dimensions—symptoms, physical activity capacity, and disease impact—with 35 items in total. Each item is rated on a 0–3 scale based on severity, resulting in a total score ranging from 0 to 100 points, where higher scores indicate poorer quality of life. Patients completed the questionnaire independently before intervention and at 1, 2, and 4 weeks postintervention.

#### 2.3.4. Safety and Efficacy Indicators

(1) Adverse events: Calculate the incidence of hemoptysis (blood‐tinged sputum ≥ 5 mL), pneumothorax (confirmed by imaging), and infection spread (new pulmonary infiltrates or existing infiltrates expanding) within 4 weeks of intervention. (2) Total interventional efficacy rate: Based on symptom, physical sign, and imaging assessment, significant efficacy is defined as marked improvement in coughing and sputum production with complete resolution of purulent sputum and minimal lung rales, along with imaging evidence of substantial inflammation absorption; effective: symptom alleviation with reduced purulent sputum and decreased lung rales, showing partial inflammation absorption on imaging; ineffective: worsening symptoms without improvement or expanding inflammation on imaging. Total efficacy eate = (number of significant efficacy cases + number of effective cases)/total cases × 100%.

### 2.4. Statistical Methods

Statistical analysis was conducted using SPSS25.0 software. For normally distributed quantitative data, mean values ± standard deviation were presented, with *t*‐tests employed for intergroup comparisons. Categorical data were expressed as frequency and percentage (%), with *χ* ^2^‐tests applied for group comparisons. Rank data were analyzed using rank sum tests. The dynamic effects of intervention methods on relevant indicators were examined through generalized estimation equations (GEEs), utilizing exchangeable correlation matrices, which appropriately account for the within‐subject correlation of repeated measurements over time. Statistical significance was defined as *p* < 0.05.

## 3. Results

### 3.1. Comparison of General Data Between the Two Groups

As shown in Table 1, there was no significant difference between the two groups in age, gender, disease course, lesion site, and basic disease (*p* > 0.05), which were comparable. The underlying etiologies of bronchiectasis in both groups included postinfectious (intervention group: 22 [55.0%] vs. control group: 20 [50.0%]), idiopathic (10 [25.0%] vs. 12 [30.0%]), and immunodeficiency‐related (8 [20.0%] vs. 8 [20.0%]), with no significant difference between groups (*p* > 0.05). Respiratory comorbidities including asthma (intervention: 5 [12.5%] vs. control: 4 [10.0%]) and COPD (intervention: 6 [15.0%] vs. control: 7 [17.5%]) were comparable between groups (*p* > 0.05). The Reiff score for bronchiectasis severity was 7.2 ± 3.1 in the intervention group and 6.8 ± 2.9 in the control group (*p* = 0.542). The distribution of bronchiectasis types (cylindrical, varicose, and cystic) and the number of affected lung lobes were also similar between groups (*p* > 0.05).

**TABLE 1:** Comparison of clinical characteristics between the two groups [(*x* ± *s*), *n* (%)].

Indicator	Observation group (*n* = 40)	Control group (*n* = 40)	t/x^2^	*p*
Age (years)	56.38 ± 10.25	55.72 ± 11.43	0.272	0.786
Gender				
Male	23 (57.50)	21 (52.50)	0.202	0.653
Female	17 (42.50)	19 (47.50)		
Course of disease (years)	8.76 ± 3.42	9.15 ± 3.78	0.484	0.630
Lesion location				
Left lung	15 (37.50)	14 (35.00)	0.065	0.968
Right lung	16 (40.00)	17 (42.50)		
Both lungs	9 (22.50)	9 (22.50)		
Basic diseases				
Hypertension	12 (30.00)	10 (25.00)	0.251	0.617
Diabetes mellitus	8 (20.00)	9 (22.50)	0.075	0.785
Coronary heart disease	5 (12.50)	6 (15.00)	0.105	0.745

### 3.2. Comparison of Pulmonary Function Indices Between the Two Groups

**FIGURE 1:**
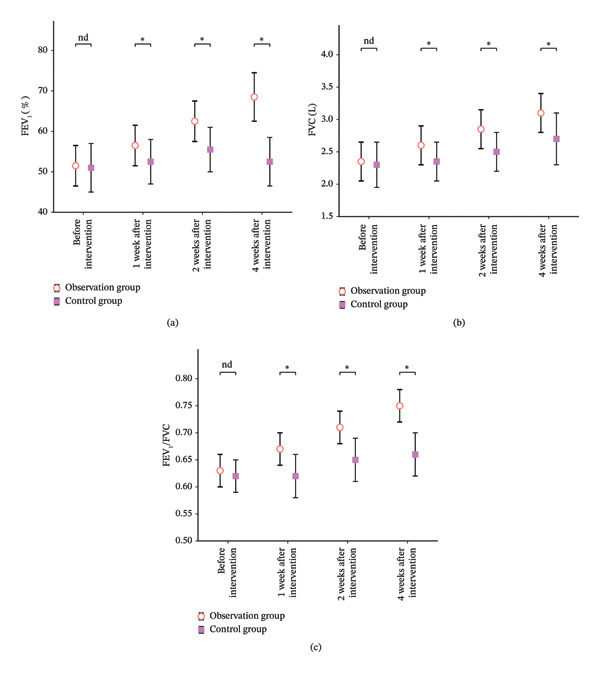
Comparison of bar charts of two groups of pulmonary function indicators. Note: Figure 1 (a)–(c), respectively, correspond to the comparison of differences of two groups of pulmonary function indices at different time points, where “nd” represents *p* > 0.05 and “^∗^” represents *p* < 0.05.

As shown in Table 2, there was no significant difference in the comparison of lung function indices between the two groups before intervention (*p* > 0.05); after 1 week, 2 weeks, and 4 weeks of intervention, all indices of the two groups improved compared with that before intervention, and the intervention group was significantly higher than that of the control group (*p* < 0.05) (Figure 1).

**TABLE 2:** Comparison of pulmonary function indices between the two groups (*x* ± *s*).

Indicator	Group	*n*	Before intervention	1 week after intervention	2 weeks after intervention	4 weeks after intervention
FEV1 (%)	Observation group	40	51.27 ± 6.38	56.43 ± 5.82	62.15 ± 5.47	68.43 ± 5.72
	Control group	40	50.83 ± 7.15	52.36 ± 6.25	55.72 ± 6.03	52.17 ± 6.38
	*t*		0.290	3.014	4.995	12.001
	*p*		0.772	0.003	0.000	0.000

FVC (L)	Observation group	40	2.35 ± 0.42	2.58 ± 0.38	2.87 ± 0.35	3.12 ± 0.32
	Control group	40	2.31 ± 0.45	2.39 ± 0.41	2.56 ± 0.39	2.68 ± 0.43
	*t*		0.411	2.150	3.741	5.192
	*p*		0.682	0.035	0.000	0.000

FEV1/FVC	Observation group	40	0.63 ± 0.05	0.67 ± 0.04	0.71 ± 0.04	0.75 ± 0.03
	Control group	40	0.62 ± 0.06	0.63 ± 0.05	0.65 ± 0.05	0.66 ± 0.04
	*t*		0.810	3.951	5.926	11.384
	*p*		0.421	0.000	0.000	0.000

*Note:* FEV1: percentage of the predicted value of forced expiratory volume in the first second; FEV1/FVC: forced expiratory volume in the first second/forced vital capacity (一秒率).

Abbreviation: FVC, forced vital capacity.

### 3.3. Comparison of Inflammatory Indicators Between the Two Groups

**FIGURE 2:**
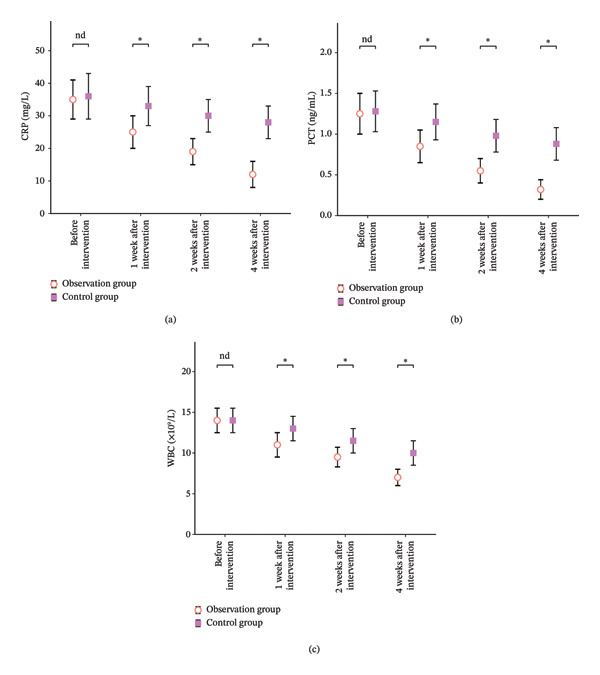
Comparison of two groups of inflammatory indicators bar chart. Note: Figure 2 (a)–(c), respectively, show the difference of two groups of inflammatory indices at different time points, where “nd” represents *p* > 0.05 and “^∗^” represents *p* < 0.05.

As shown in Table 3, there was no statistically significant difference between the two groups before intervention (*p* > 0.05). After 1 week, 2 weeks, and 4 weeks of intervention, all indicators in the intervention group were significantly lower than those before intervention, and significantly lower than those in the control group at the same time (*p* < 0.05) (Figure 2).

**TABLE 3:** Comparison of pulmonary function indices between the two groups (*x* ± *s*).

Indicator	Group	*n*	Before intervention	1 week after intervention	2 weeks after intervention	4 weeks after intervention
CRP (mg/L)	Observation group	40	35.62 ± 6.84	25.37 ± 5.26	18.43 ± 4.12	12.38 ± 3.25
	Control group	40	36.15 ± 7.23	32.46 ± 6.15	29.72 ± 5.87	27.64 ± 4.81
	*t*		0.337	5.541	9.957	16.626
	*p*		0.737	0.000	0.000	0.000

PCT (ng/mL)	Observation group	40	1.25 ± 0.32	0.87 ± 0.25	0.56 ± 0.18	0.32 ± 0.11
	Control group	40	1.28 ± 0.35	1.12 ± 0.31	0.98 ± 0.26	0.87 ± 0.23
	*t*		0.400	3.970	8.400	13.644
	*p*		0.690	0.000	0.000	0.000

WBC (× 10^9^/L)	Observation group	40	13.65 ± 2.18	11.23 ± 1.87	9.42 ± 1.56	6.84 ± 1.23
	Control group	40	13.92 ± 2.35	12.87 ± 2.05	11.65 ± 1.82	9.76 ± 1.58
	*t*		0.533	3.738	5.884	9.223
	*p*		0.596	0.000	0.000	0.000

*Note:* PCT: procalcitonin.

Abbreviations: CRP, C‐reactive protein; WBC: white blood cell count.

### 3.4. Comparison of SGRQ Scores Between the Two Groups

**FIGURE 3:**
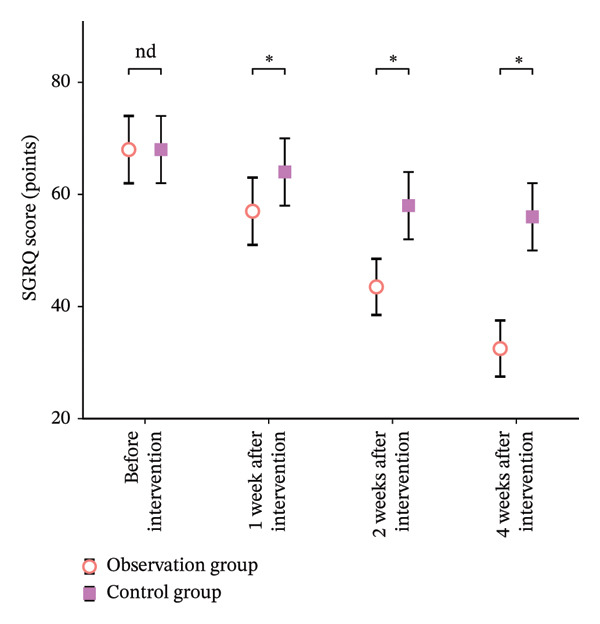
Comparison bar chart of scores from two groups of St George’s Respiratory Questionnaire. Note: “nd” means *p* > 0.05 and “^∗^” means *p* < 0.05.

As shown in Table 4, there was no significant difference between the two groups before intervention (*p* > 0.05). After 1 week, 2 weeks, and 4 weeks of intervention, the scores of both groups decreased compared with that before intervention, and the intervention group was significantly lower than that of the control group (*p* < 0.05) (Figure 3).

**TABLE 4:** Comparison of St. George’s Respiratory Questionnaire scores between the two groups (x ± s, points).

Group	*n*	Before intervention	1 week after intervention	2 weeks after intervention	4 weeks after intervention
Observation group	40	68.43 ± 7.56	57.25 ± 6.84	43.62 ± 5.73	32.57 ± 4.68
Control group	40	67.86 ± 8.12	63.57 ± 7.25	58.43 ± 6.32	56.32 ± 6.15
*t*		0.325	4.010	10.980	19.436
*p*		0.746	0.000	0.000	0.000

### 3.5. GEE Model Analyzes the Influence of Intervention Methods on Relevant Indicators

Using intervention mode (intervention group/control group), time (before intervention, 1 week, 2 weeks, and 4 weeks) and their interaction as independent variables, and each observation index as dependent variable, the GEE model was constructed. The data were fitted by exchange correlation matrix. The results showed the following.

In pulmonary function parameters, the main effects of the intervention group showed significant time‐dependent improvements: FEV1 percentage of predicted value *β* = 5.381 (*p* < 0.001), time *β* = 3.764 (*p* < 0.001), and interaction *β* = 3.273 (*p* < 0.001). The intervention group demonstrated greater improvement over time. For FVC, the main effect *β* = 0.294 (*p* = 0.009), time *β* = 0.183 (*p* < 0.001), and interaction *β* = 0.214 (*p* = 0.001) all indicated a progressively stronger advantage with time. In FEV1/FVC ratio, the main effect *β* = 0.042 (*p* < 0.001) and interaction *β* = 0.043 (*p* < 0.001) suggested potential enhancement of expiratory efficiency.

In the inflammatory markers analysis, the CRP intervention group showed significant main effects: *β* = −8.452 (*p* < 0.001), time main effect *β* = −4.083 (*p* < 0.001), and interaction *β* = −4.721 (*p* < 0.001), indicating that anti‐inflammatory effects accumulate over time. The PCT intervention group demonstrated stronger anti‐infection efficacy with main effect *β* = −0.432 (*p* < 0.001) and interaction *β* = −0.223 (*p* = 0.001). The leukocyte count intervention group exhibited a more persistent infection control effect with main effect *β* = −2.213 (*p* < 0.001) and interaction *β* = −1.372 (*p* = 0.002), showing significant advantages in suppressing inflammation.

The main effect of SGRQ score intervention group was *β* = −12.763 (*p* < 0.001), and the interaction effect was *β* = −8.832 (*p* < 0.001). The advantage of quality of life improvement increased with time (Table 5).

**TABLE 5:** Results of generalized estimating equation (GEE) analysis for related indicators.

Indicator	Variable	*β*	*SE*	Wald*χ* ^2^	*p*
FEV1 (%)	(Intercept)	50.972	1.324	1486.732	< 0.001
	Observation group	5.381	1.152	21.643	< 0.001
	Control group	0.000^∗^	—	—	—
	Time	3.764	0.582	41.891	< 0.001
	Time × Observation group	3.273	0.691	22.412	< 0.001
	Time × Control group	0.000^∗^	—	—	—

FVC(L)	(Intercept)	2.321	0.093	684.524	< 0.001
	Observation group	0.294	0.112	6.872	0.009
	Control group	0.000^∗^	—	—	—
	Time	0.183	0.041	19.635	< 0.001
	Time × Observation group	0.214	0.061	12.253	0.001
	Time × Control group	0.000^∗^	—	—	—

FEV1/FVC	(Intercept)	0.623	0.01	3844	< 0.001
	Observation group	0.042	0.011	16.002	< 0.001
	Control group	0.000^∗^	—	—	—
	Time	0.031	0.01	9.003	0.003
	Time × Observation group	0.043	0.011	16.002	< 0.001
	Time × Control group	0.000^∗^	—	—	—

CRP (mg/L)	(Intercept)	35.792	1.613	497.261	< 0.001
	Observation group	−8.452	1.721	24.183	< 0.001
	Control group	0.000^∗^	—	—	—
	Time	−4.083	0.832	23.812	< 0.001
	Time × Observation group	−4.721	0.973	23.651	< 0.001
	Time × Control group	0.000^∗^	—	—	—

PCT (ng/mL)	(Intercept)	1.271	0.092	198.892	< 0.001
	Observation group	−0.432	0.101	18.492	< 0.001
	Control group	0.000^∗^	—	—	—
	Time	−0.241	0.06	16.001	<a00.001
	Time × Observation group	−0.223	0.07	10.293	0.001
	Time × Control group	0.000^∗^	—	—	—

WBC (× 10^9^/L)	(Intercept)	13.821	0.472	876.163	< 0.001
	Observation group	−2.213	0.581	14.522	< 0.001
	Control group	0.000^∗^	—	—	—
	Time	−1.852	0.361	26.032	< 0.001
	Time × Observation group	−1.372	0.451	9.292	0.002
	Time × Control group	0.000^∗^	—	—	—

SGRQ score	(Intercept)	68.052	1.932	1239.472	< 0.001
	Observation group	−12.763	2.281	31.242	< 0.001
	Control group	0.000^∗^	—	—	—
	Time	−8.272	1.312	40.061	< 0.001
	Time × Observation group	−8.832	1.613	30.122	< 0.001
	Time × Control group	0.000^∗^	—	—	—

*Note:* 0^∗^ indicates that the parameter is a reference value and is set to 0.

Abbreviation: SGRQ score, St. George’s Respiratory Questionnaire score.

While the GEE model captures the time‐dependent trend and the progressive advantage of the intervention, independent‐samples *t*‐tests at the final 4‐week timepoint confirmed significant between‐group differences in absolute values: FEV1% predicted was significantly higher in the intervention group than in the control group (*p* < 0.05), and CRP, PCT, and SGRQ scores were significantly lower in the intervention group (*p* < 0.05), as shown in Tables 2, 3, and 4. Thus, the intervention group demonstrated superiority both in the trajectory of improvement and in the final achieved values.

### 3.6. Microbiological Findings From Bronchoscopy

Among the 40 patients in the intervention group, bronchoscopic lavage specimens yielded positive cultures in 32 cases (80.0%). The most commonly isolated pathogens were *Pseudomonas aeruginosa* (*n* = 12, 30.0%), Haemophilus influenzae (*n* = 8, 20.0%), Streptococcus pneumoniae (*n* = 5, 12.5%), and fungal organisms (*n* = 4, 10.0%). In 18 cases (45.0%), the bronchoscopic culture results led to modification of the initial empirical antibiotic regimen, including escalation to antipseudomonal agents in 10 cases and addition of antifungal therapy in 3 cases. The identification of specific pathogens through bronchoscopy enabled more targeted antimicrobial therapy, which may have contributed to the superior clinical outcomes observed in the intervention group.

### 3.7. Comparison of Adverse Events and Efficacy Between the Two Groups

**FIGURE 4:**
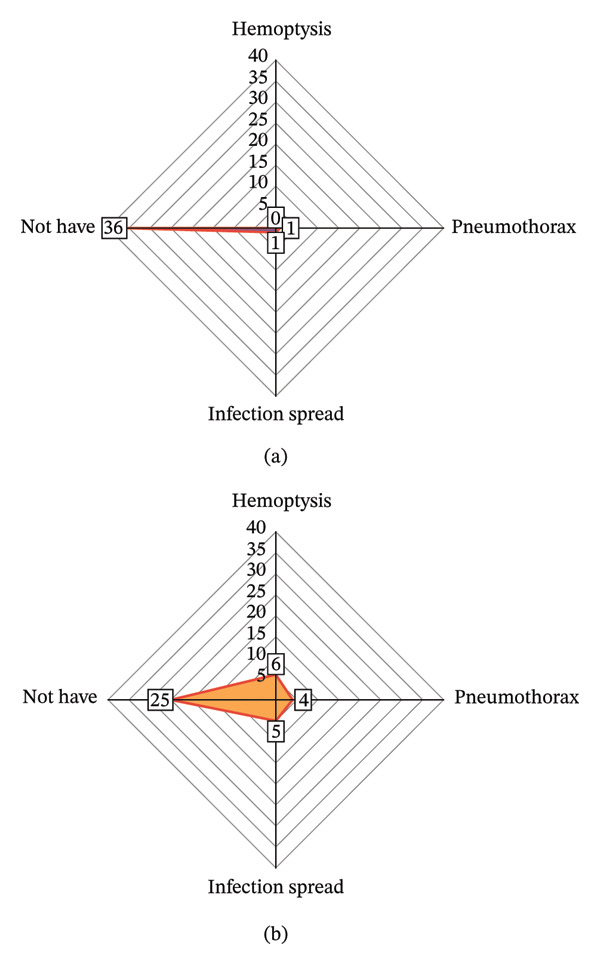
Comparison radar chart of adverse events between the two groups. Note: Figure 4 (a, b) are the radar charts of adverse events in the intervention group and control group, respectively.

**FIGURE 5:**
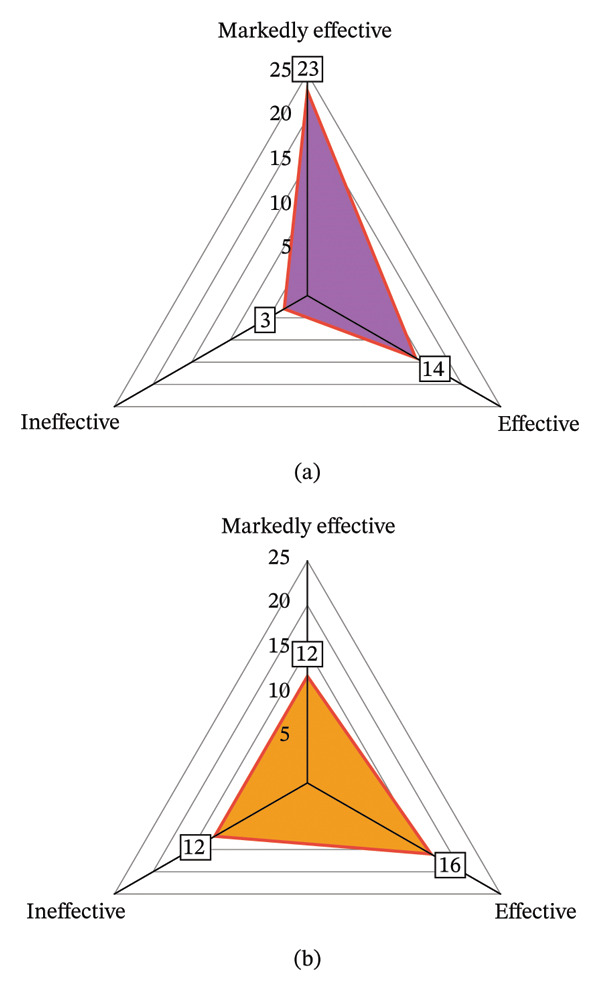
Two sets of efficacy radar charts. *Note*: Figure 5 (a, b) correspond to the efficacy radar chart of the intervention group and control group, respectively.

The total incidence of adverse events in the intervention group was significantly lower than that in the control group (*p* < 0.05); the total effective rate of intervention in the intervention group was significantly higher than that in the control group (*p* < 0.05) (Table 6 and Figures 4 and 5).

**TABLE 6:** Comparison of adverse reactions and efficacy between the two groups [*n* (%)].

Indicator	Category	Observation group (*n* = 40)	Control group (*n* = 40)	*x* ^2^ */Z*	*p*
Adverse reactions	Hemoptysis	2 (5.00)	6 (15.00)	8.352	0.004
	Pneumothorax	1 (2.50)	4 (10.00)		
	Infection spread	1 (2.50)	5 (12.50)		
	Total incidence rate	4 (10.00)	15 (37.50)		

Intervention effect	Markedly effective	23 (57.50)	12 (30.00)	2.905	0.004
	Effective	14 (35.00)	16 (40.00)		
	Ineffective	3 (7.50)	12 (30.00)		
	Total effective rate	37 (92.50)	28 (70.00)	6.646	0.010

Regarding direct procedure‐related adverse events in the intervention group: transient minor hemoptysis (< 5 mL) occurred in 3 patients (7.5%), which resolved spontaneously or with oral Yunnan Baiyao within 24 h; transient oxygen desaturation during the procedure (SpO2 < 90% for < 2 min) was observed in 2 patients (5.0%), managed by brief supplemental oxygen; postprocedure sore throat was reported in 5 patients (12.5%), resolving within 48 h without treatment; and transient fever (> 38.0°C within 24 h postprocedure) occurred in 2 patients (5.0%), managed with antipyretics. No cases of pneumothorax, significant hemorrhage (> 10 mL), or procedure‐related mortality occurred. All procedure‐related adverse events were mild and self‐limiting.

## 4. Discussion

The core pathophysiological feature of acute exacerbation in bronchiectasis is the pathogenetic vicious vortex of airway infection, bronchial structural distortion, mucociliary clearance impairment, and immune dysregulation. The retention of purulent secretions in airways not only exacerbates local infection spread but also leads to progressive decline in pulmonary function through mechanical obstruction and inflammatory factor release [12, 13]. This study innovatively combined chest HRCT imaging assessment with bronchoscopic interventional techniques. Results demonstrated that this combined approach significantly outperformed conventional interventions in improving pulmonary function, controlling inflammation, and enhancing quality of life, while offering greater safety. This provides a precise solution to break the aforementioned vicious vortex.

From the perspective of pulmonary function improvement mechanisms, the intervention group demonstrated significant advantages as early as 1 week posttreatment, with these benefits progressively expanding over time. The HRCT 3D reconstruction technology enables precise localization of structural obstructions such as mucus plugs and strictures, transitioning bronchoscopy from experience‐dependent techniques to anatomy‐guided procedures that avoid damage to normal airways caused by traditional blind visualization [8, 9]. During surgery, differentiated clearance strategies were implemented for different grades of mucus plugs (using biopsy forceps for Grades I‐II mucus plugs and cryoprobe treatment for Grade III hard mucus plugs), combined with staged irrigation and localized antibiotic administration, effectively resolving airway obstruction while reducing bacterial load [14]. GEE model analysis revealed that improvements in FEV1, FVC, and FEV1/FVC showed significant progression over time in the intervention group, indicating that this technique not only provides short‐term relief of airway obstruction but also achieves sustained pulmonary function optimization by mitigating chronic inflammatory damage to airway walls. Importantly, while the GEE model captures the temporal trend of progressive improvement, independent between‐group comparisons at the final 4‐week timepoint confirmed that the intervention group achieved significantly higher absolute FEV1 values, demonstrating superiority in both trajectory and final outcome.

The dynamic changes in inflammatory markers further validated the advantages of this technology. In the intervention group, CRP, PCT, and WBC levels were significantly lower than those in the control group after just 1 week of intervention, with the reduction rate increasing over time. This outcome is closely related to the dual effects of localized intervention: On one hand, airway clearance reduces the source of bacterial toxin and inflammatory mediator release [15]; on the other hand, precise delivery of locally sensitive antibiotics can rapidly eliminate pathogens at high concentrations, avoiding the “subtherapeutic concentration” issue caused by insufficient tissue penetration from systemic administration [16]. Studies indicate that antibiotic concentrations at infection sites in bronchiectasis patients need to reach 5–10 times the blood concentration to effectively clear bacteria encased in biofilms. The targeted drug delivery achieved through HRCT localization precisely meets this requirement, which explains why the intervention group demonstrated more sustained inflammation control.

An important added value of bronchoscopy in this study was the enhanced pathogen identification through direct bronchoscopic sampling. In our intervention group, bronchoscopic lavage yielded positive cultures in 80.0% of patients, with *Pseudomonas aeruginosa* being the most frequently isolated pathogen (30.0%). This is particularly relevant given that Pseudomonas colonization is associated with worse outcomes in bronchiectasis. A recent multicenter cohort study of patients with hematology‐related bronchiectasis demonstrated significantly higher rates of Pseudomonas isolation and worse prognosis in immunocompromised populations, highlighting the potential importance of bronchoscopic intervention for pathogen identification and targeted antimicrobial therapy in these patients [17]. The modification of antibiotic regimens in 45.0% of our bronchoscopy patients based on culture results underscores the clinical value of this procedure beyond mechanical airway clearance, as targeted therapy may itself contribute to the superior outcomes observed.

It should be noted that the observed clinical improvements are more likely attributable to the bronchoscopic procedure itself rather than solely to transient sputum clearance, as secretions can reaccumulate rapidly after the procedure. The bronchoscopy provided multiple therapeutic benefits beyond mechanical clearance: (1) precise pathogen identification through direct sampling, enabling targeted antibiotic therapy in 45.0% of cases; (2) localized delivery of high‐concentration antibiotics directly to the infection site, achieving tissue concentrations far exceeding systemic administration; and (3) removal of mucus plugs that served as reservoirs for biofilm‐embedded bacteria. These combined mechanisms likely account for the sustained improvements observed over the 4‐week follow‐up period.

The improvement in quality of life serves as a comprehensive indicator of treatment efficacy. The SGRQ scores demonstrated that the intervention group showed greater improvements than the control group across three dimensions: symptom reduction, activity capacity, and disease impact, with nearly 50% score decrease observed at 4‐week follow‐up compared with baseline levels. This outcome stems not only from enhanced pulmonary function alleviating dyspnea but also directly correlates with reduced coughing and sputum production following mucus clearance [18, 19]. Notably, the intervention group exhibited significantly lower overall adverse event rates (10.00% vs. 37.50%), attributed to two key factors: HRCT‐guided procedures avoiding unnecessary access to high‐risk lesions (e.g., bronchi with severe dilation and vascular malformations), and cryoprobe technology minimizing mucosal injury and bleeding risks [20]. Furthermore, strict control of irrigation volume (≤ 100 mL) and fluid recovery rate (≥ 50%) effectively reduces infection spread risks from extravasation into healthy lung tissue [21, 22], contrasting sharply with traditional bronchoscopy’s 25%–30% complication rate.

The innovation of this study lies in three aspects: First, it systematically validated the effectiveness of HRCT‐guided bronchoscopic intervention during acute exacerbations for the first time, addressing the previous focus on chronic stable phases. Second, through dynamic analysis using GEE models, we confirmed that the therapeutic benefits are sustained rather than short term. Third, we established a graded intervention strategy based on the Reiff scoring system, providing reference for individualized management of lesions with varying severity levels. However, several limitations should be acknowledged: First, this study was not prospectively registered in an international trial registry, which limits transparency. Second, the small sample size and single‐center design may affect result extrapolation. Third, subgroup analyses were not conducted to evaluate intervention response differences among pathogens (e.g., *Pseudomonas aeruginosa* and Haemophilus influenzae). Fourth, long‐term follow‐up data are lacking to assess the technology’s impact on acute exacerbation frequency and annual decline rate of lung function. Fifth, the relatively small standard deviations observed in our outcome measures, particularly for inflammatory markers such as CRP, may appear unusual given the known variability of these parameters. We have reverified all data against the original medical records and confirm their accuracy. The narrow SDs likely reflect the strict inclusion criteria (FEV1 ≤ 30% predicted, specific exacerbation definition requiring ≥ 3 symptoms for ≥ 48 h), which selected a homogeneous population with similar baseline disease severity. Additionally, the single‐center design with standardized measurement protocols and uniform laboratory methods may have further reduced interpatient variability. We acknowledge that this homogeneity may limit external generalizability, and future multicenter studies with larger, more heterogeneous populations are needed. Sixth, the general SGRQ was used for quality‐of‐life assessment instead of the bronchiectasis‐specific Quality of Life‐Bronchiectasis (QOL‐B) tool, which may have been more sensitive to disease‐specific changes. Seventh, only the intervention group underwent HRCT, which may have influenced clinical decision‐making beyond the bronchoscopic procedure itself. Eighth, the total duration of hospitalization and time to next acute exacerbation were not evaluated as outcomes, which are important endpoints in bronchiectasis management. Future research should include multicenter, large‐sample cohort studies combined with microbiome analysis to further optimize intervention protocols.

## 5. Conclusion

In summary, image‐assisted bronchoscopic interventions significantly improve clinical outcomes in patients with acute exacerbations of bronchiectasis. By precisely relieving airway obstruction, targeting infection control, and reducing inflammatory responses, this technique demonstrates excellent safety profiles. As a scalable precision intervention model that breaks the pathogenetic vicious vortex, it warrants further clinical implementation and promotion.

**Nomenclature**
HRCT: High‐resolution computed tomography
FEV_1_
: Forced expiratory volume in the first second
FVC: Forced vital capacity
FEV1/FVC: Ratio of forced expiratory volume in the first second to forced vital capacity
CRP: C‐reactive protein
PCT: Procalcitonin
WBC: White blood cell count
SGRQ: St. George’s Respiratory Questionnaire
GEEs: Generalized estimation equations
MPR: Multiplanar reconstruction
VR: Volumetric reconstruction
mMRC: modified Medical Research Council (dyspnea scale)
PA: Pulmonary artery

## Author Contributions

Biliang Li and Zeng Juan contributed to the conception and design of the study; Mingfeng Wang and Wei Kang performed the experiments and collected and analyzed data; Biliang Li wrote the manuscript; and Bo Li revised the manuscript.

## Funding

No funding was received for this study.

## Disclosure

All authors have reviewed and approved the final version of the manuscript.

## Ethics Statement

The current study was conducted in accordance with the Helsinki Declaration of the World Medical Association and approved by the Ethics Committee of our hospital (approval no. 2019–086), with all patients or their legal guardians signing written informed consent.

## Consent

Please see the Ethics Statement.

## Conflicts of Interest

The authors declare no conflicts of interest.

## Data Availability

The datasets analyzed during the current study are not publicly available due to the personal privacy but are available from the corresponding author upon reasonable request.
